# Elevated circulating level of β-aminoisobutyric acid (BAIBA) in heart failure patients with type 2 diabetes receiving sodium-glucose cotransporter 2 inhibitors

**DOI:** 10.1186/s12933-022-01727-x

**Published:** 2022-12-20

**Authors:** Satoshi Katano, Toshiyuki Yano, Hidemichi Kouzu, Ryohei Nagaoka, Ryo Numazawa, Kotaro Yamano, Yusuke Fujisawa, Katsuhiko Ohori, Nobutaka Nagano, Takefumi Fujito, Ryo Nishikawa, Wataru Ohwada, Masaki Katayose, Tatsuya Sato, Atsushi Kuno, Masato Furuhashi

**Affiliations:** 1 Division of Rehabilitation, Sapporo Medical University Hospital, South-1, West-16, Chuo-ku, Sapporo, 060-8543 Japan; 2 Department of Cardiovascular, Renal and Metabolic Medicine, Sapporo Medical University School of Medicine, South-1, West-16, Chuo-ku, Sapporo, 060-8543 Japan; 3 Graduate School of Medicine, Sapporo Medical University, South-1, West-16, Chuo-ku, Sapporo, 060-8543 Japan; 4 Department of Cardiology, Hokkaido Cardiovascular Hospital, Sapporo, Japan; 5 Second Division of Physical Therapy, Sapporo Medical University School of Health Science, South-1, West-16, Chuo-ku, Sapporo, 060-8543 Japan; 6 Department of Cellular Physiology and Signal Transduction, Sapporo Medical University School of Medicine, South-1, West-16, Chuo-ku, Sapporo, 060-8543 Japan; 7 Department of Pharmacology, Sapporo Medical University School of Medicine, South-1, West-16, Chuo-ku, Sapporo, 060-8543 Japan

**Keywords:** Heart failure, Diabetes mellitus, Amino acid, β-aminoisobutyric acid, Sodium-glucose cotransporter 2 inhibitors, SGLT2

## Abstract

**Aims:**

The mechanism by which a sodium-glucose cotransporter inhibitor (SGLT2i) induces favorable effects on diabetes and cardiovascular diseases including heart failure (HF) remains poorly understood. Metabolomics including amino acid profiling enables detection of alterations in whole body metabolism. The aim of this study was to determine whether plasma amino acid profiles are modulated by SGLT2i use in HF patients with type 2 diabetes mellitus (T2DM).

**Methods:**

We retrospectively examined 81 HF patients with T2DM (68 ± 11 years old; 78% male). Plasma amino acid concentrations in a fasting state after stabilization of HF were determined using ultraperformance liquid chromatography. To minimize potential selection bias in the retrospective analyses, the differences in baseline characteristics between patients receiving an SGLT2i and patients not receiving an SGLT2i were controlled by using an inverse probability of treatment weighting (IPTW)-adjusted analysis.

**Results:**

Of amino acids measurable in the present assay, plasma β﻿﻿-aminoisobutyric acid (BAIBA), an exercise-induced myokine-like molecule also known as 3-aminoisobutyric acid or 3-amino-2-methyproponic acid, was detected in 77% of all patients and the proportion of patients in whom plasma BAIBA was detected was significantly higher in patients receiving an SGLT2i than in patients not receiving an SGLT2i (93% vs. 67%, p = 0.01). Analyses in patients in whom plasma BAIBA was detected showed that plasma BAIBA concentration was significantly higher in patients receiving an SGLT2i than in patients not receiving an SGLT2i (6.76 ± 4.72 vs. 4.56 ± 2.93 nmol/ml, p = 0.03). In multivariate logistic regression analyses that were adjusted for age and sex, SGLT2i use was independently associated with BAIBA detection. The independent association between BAIBA and SGLT2i use remained after inclusion of body mass index, HF with reduced ejection fraction, ischemic etiology, renal function, NT-proBNP, albumin, hemoglobin, and HbA1c into the Cox proportional hazards model. When the differences in baseline characteristics between patients receiving an SGLT2i and patients not receiving an SGLT2i were controlled by using an IPTW-adjusted analysis, least squares mean of plasma BAIBA concentration was significantly higher in patients receiving an SGLT2i than in patients not receiving an SGLT2i.

**Conclusion:**

SGLT2i use is closely associated with increased circulating BAIBA concentration in HF patients with T2DM.

## Background

Heart failure (HF) is a major public health problem with a current estimated prevalence of approximately 60 million worldwide, and it is a leading cause of morbidity and mortality, though there have been improvements due to development of treatment strategies [1, 2]. Sodium-glucose cotransporter 2 (SGLT2) inhibitors (SGLT2i) are a novel class of oral glucose-lowering agents that work by inhibiting SGLT2, a protein that is located in the early proximal tubule of the nephron and is responsible for the reabsorption of approximately 90% of filtered glucose, leading to glycosuria together with natriuresis [3–5]. Glycosuria by SGLT2 inhibition results in reduction in plasma glucose concentrations in addition to improvements in overweight, elevated blood pressure, lipid profile, and hyperuricemia. Results of recent clinical studies have repeatedly showed that treatment with an SGLT2i reduces cardiovascular events in patients with type 2 diabetes mellitus (T2DM) [3–6]. Such a clinical benefit has been seen in HF patients without T2DM [7, 8], indicating that the cardioprotective actions of SGLT2i are not solely explainable by improvement in glycemic control. Intriguingly, the impact of an SGLT2i on favorable outcome is mainly attributable to prominent reduction in HF hospitalization and preserved renal function [9, 10]. A similar benefit for HF hospitalization has been found in patients who have HF reduced ejection fraction (HFrEF) and patients who have HF preserved ejection fraction (HFpEF) [11, 12]. Therefore, an SGLT2i is a cornerstone drug for HF treatment independently of the patient’s background. However, the mechanism by which an SGLT2i induces favorable effects on cardiovascular diseases and chronic kidney disease remains poorly understood, though several possibilities including off-target effects such as effects on sodium/hydrogen exchanger and/or sodium channel (Nav1.5) have been proposed [13, 14].

Amino acids serve as building blocks of proteins and also metabolic intermediates in the regulation of multiple cell functions. In addition, non-proteinogenic amino acids play a pivotal role in intermediates in physiological processes and pathogenesis of diseases and their plasma levels are modulated by sensing alterations in whole body metabolism. Therefore, plasma amino acid profiling is a useful approach for elucidating the pathogenesis of disease and response to treatment, as is omics [15–19]. Indeed, the results of our recent study showed that specific patterns of changes in circulating amino acid concentrations improved the predictive ability for adverse events [20], indicating the utility of plasma amino acid profiling in risk stratification of HF patients. Intriguingly, urinary amino acid concentrations have been shown to be modulated by genetic and pharmacological inhibition of SGLT2 [21, 22], possibly leading to changes in plasma amino acid profiles. In addition, results of our metabolomic analyses using rat hearts revealed that treatment with empagliflozin, an SGLT2i, had profound impacts on cardiac metabolites including amino acids [23, 24]. These findings led us to examine alteration in the circulating amino acid profile by SGLT2 inhibition in HF patients to unveil the mechanism of SGLT2i-mediated cardioprotection.

In the present study, systematic analyses of the relationships between SGLT2i use and plasma amino acid concentrations in HF patients with T2DM were performed. To minimize potential selection bias in the retrospective analyses, the differences in baseline characteristics between patients receiving an SGLT2i and patients not receiving an SGLT2i were controlled by using an inverse probability of treatment weighting (IPTW)-adjusted analysis.

## Methods

### Study subjects

**Fig. 1:**
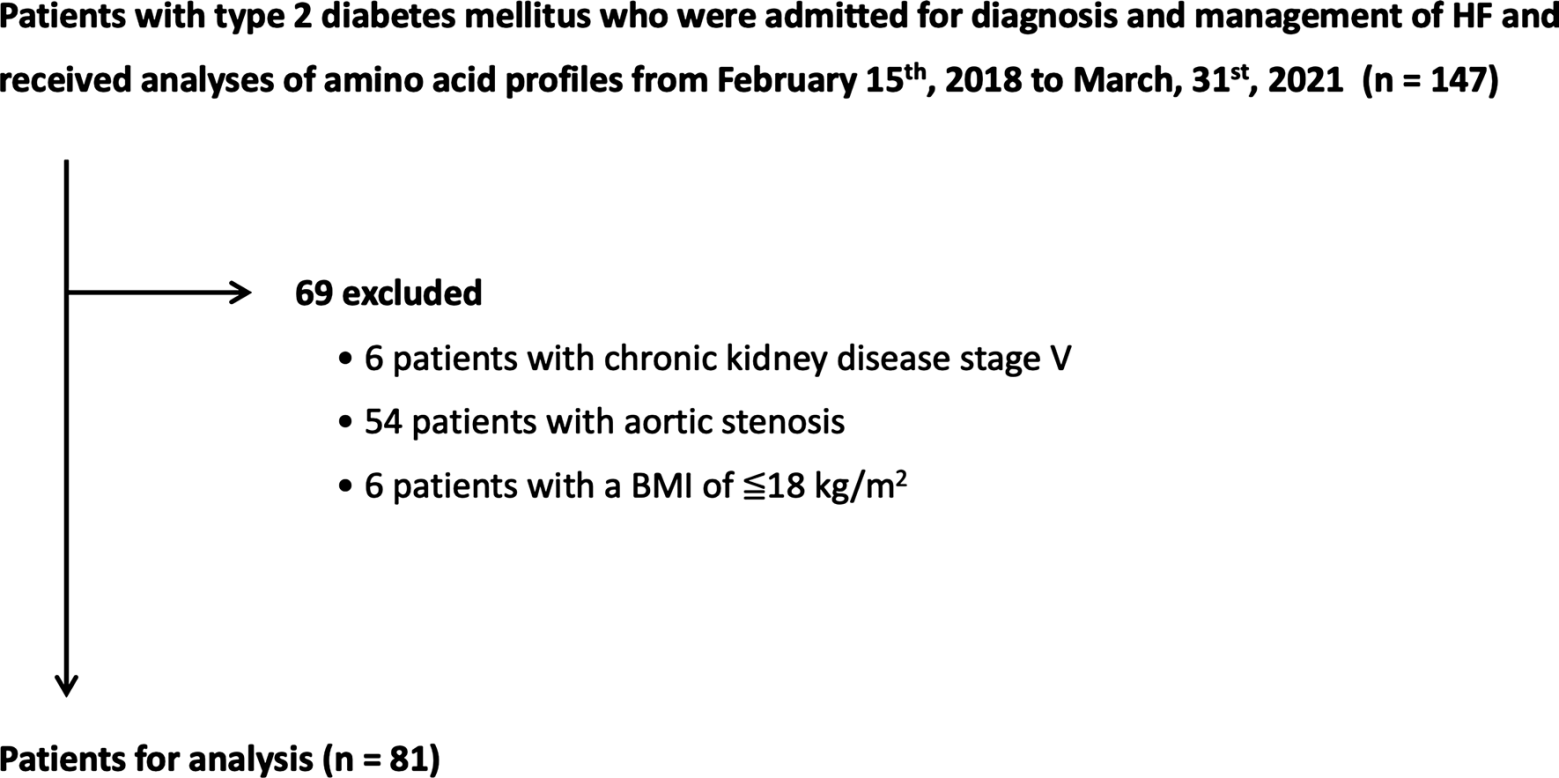
Flow chart of inclusion of patients. HF, heart failure; BMI, body mass index

This study was a single-center, retrospective, and observational study. We retrospectively enrolled consecutive patients with T2DM who were admitted to our institute for diagnosis and management of HF during the period from February 15th, 2018 to March 31st, 2020. The inclusion criterion was diagnosis of HF according to the 2016 ESC Guidelines for the diagnosis and treatment of acute and chronic heart failure [25]. Exclusion criteria were pulmonary artery hypertension, aortic stenosis, and chronic kidney disease at stage 5 that was defined as estimated glomerular filtration rate (eGFR) < 15 ml/min/1.73 m^2^, as shown in Fig. 1. Patients with body mass index (BMI) being < 18 kg/m^2^ were also excluded since SGLT2i use is avoided in patients with low BMI. This study was conducted in strict adherence with the principles of the Declaration of Helsinki and was approved by the Clinical Investigation Ethics Committee of Sapporo Medical University Hospital (Number 302–243).

### Biochemical analyses, echocardiography, body composition analyses and assessment of nutritional status

Data for blood tests including N-terminal pro-brain natriuretic peptide (NT-proBNP) were retrieved from the patients’ medical records. Creatinine-based eGFR (eGFRcre) and cystatin C-based eGFR (eGFRcys) were calculated using equations developed for Japanese subjects as follows: eGFRcre (ml/min/1.73m^2^) = 194×Creatinine^− 1.094^×age^− 0.287^(×0.739 if female) and eGFRcys (ml/min/1.73m^2^) = 104×Cystatin C^− 1.019^ × 0.996^age^(×0.929 if female)− 8 [26]. Transthoracic echocardiography was performed by the standard protocol, and the left ventricular ejection fraction (LVEF) was measured by the modified Simpson method.

### Measurement of plasma amino acid concentration

After stabilization of HF, blood samples for measurements of amino acid concentrations were collected in a fasting state. Plasma amino acid concentrations were measured using ultraperformance liquid chromatography (LSI Medience Corporation, Tokyo, Japan) as previously described [20, 27, 28]. Blood samples were collected in sodium heparin tubes (NIPRO, Osaka, Japan) and transported to the laboratory at room temperature. Plasma was separated from whole blood by centrifugation at 3500 rpm for 7 min. Samples frozen at − 10 ^o^C were transported to LSI Medience Corporation within a few days for subsequent analyses. Ultraperformance liquid chromatography (UPLC) analysis was conducted using the Acquity™ UPLC system with TUV detector and MassTrak™ AAA Solutions Kit (Waters Corporation, Milford, MA). The MassTrak™ kit, for which the validity for clinical diagnostic applications has been evaluated in previous studies [27, 28], utilizes pre-column derivatization of amino acids with a 6-aminoquinolyl-N-hydroxysuccinimdyl carbamate followed by reversed-phase UPLC on a MassTrak AAA Column (1.7 μm; 2.1 × 150 mm) and UV detection at 260 nm. Sample preparations were conducted according to the manufacturer’s instructions with slight modifications. Briefly, plasma samples (200 µl) were deproteinized with an equal volume of 6% sulfosalicylic acid containing the internal standard Norvaline (Sigma Aldrich, St. Louis, MO) at a final concentration of 50 µmol/ml followed by centrifugation at 2130 g for 15 min at 4 ^o^C to isolate the supernatant. The deproteinized plasma samples were derivatized using the MassTrak AAA Derivatization Kit. The derivatized samples (1 µl injection volume) were separated in a column maintained at 43 ^o^C at a flow rate of 400 µl/min using the recommended solvent gradient with Eluent Buffer A (8–10% acetonitrile; 4–6% formic acid; 84–88% ammonium acetate/water) and Eluent Buffer B (> 95% acetonitrile; <5% acetic acid). In in-house validation tests conducted at LSI Medience Corporation, the limits of detection of all measurable amino acids were 1–2 nmol/ml, and intra- and interday precisions assessed by coefficients of variation were 0.7–4.0% and 3.4–7.9%, respectively, being consistent with a previous report [27]. The UPLC assay used in the present study was validated in our recent study according to the guideline developed by the Ministry of Health, Labour and Welfare of Japan (Guideline on Bioanalytical Method Validation in Pharmaceutical Development, http://www.nihs.go.jp/drug/BMV/250913_BMV-GL_E.pdf), which was produced on the basis of guidelines from the FDA and EMA [20].

Among 34 metabolites measured, metabolites detected in more than half of the patients were analyzed: 10 essential amino acids (valine, leucine, isoleucine, lysine, methionine, phenylalanine, threonine, tryptophan, histidine and tyrosine), 10 non-essential amino acids (glycine, alanine, arginine, cystine, asparagine, aspartic acid, glutamine, glutamic acid, serine, and proline), and 10 other amino acid metabolites (taurine, hydroxyproline, citrulline, β-aminoisobutyric acid [BAIBA], α-Amino-n-butyric acid, β-alanine, monoethanolamine, ornithine, 1-methylhistidine, and 3-methylhistidine). The concentrations of metabolites were normalized by auto scaling (mean-centered and divided by the standard deviation of each variable) in order to make features more comparable.

### Statistical analysis

Data are presented as means ± standard deviation or medians (interquartile range [IQR]: 25th -75th percentile) and expressed as frequency and percentage. Student’s t-test was used for a comparison of continuous variables in two groups. Differences in categorical variables between two groups were examined by the chi-square test. Multivariate logistic regression analyses were performed by incorporating variables into the baseline model (age, sex, and SGLT2i use).

To minimize the differences in potential confounding factors between patients receiving SGLT2i and patients not receiving an SGLT2i, the IPTW was calculated using propensity score (PS) [29]. A multivariate logistic regression model was fit to calculate the PS for the group receiving an SGLT2i based on the following variables: age, sex, BMI, LVEF, New York Heart Association (NYHA) functional class, eGFRcys, quartiles of NT-proBNP, levels of albumin and hemoglobin, glycated hemoglobin A1c (HbA1c), use of renin-angiotensin system inhibitors (RASi), mineralocorticoid receptor antagonists (MRA), biguanides, and dipeptidyl peptidase 4 inhibitors (DPP4i). The group receiving an SGLT2i was weighted by 1/PS, and the group not receiving an SGLT2i was weighted by 1/(1-PS). Whether covariates were balanced by the IPTW was confirmed by comparing distributions of covariates before and after IPTW using the standardized mean difference (SMD). An SMD of more than 0.1 was defined as a meaningful difference. Effects of an SGLT2i on BAIBA concentration were analyzed using generalized linear model in the IPTW-weighted cohort. Least square means of BAIBA are presented as means ± standard error.

Missing data were imputed using a multiple imputation analysis. Assuming missing at random, multiple imputations were performed using a multiple imputation by chained equations with M = 200 imputations to construct the generalized linear models using IPTW in each imputed dataset. Then the estimates were pooled to obtain the least squared means adjusted for covariates following Rubin’s rule.

A P-value < 0.05 was considered statistically significant. Statistical analyses were carried out using JMP version 15.1.0 (SAS Institute Inc., Cary, NC, USA) and R version 4.1.2 (R Foundation for Statistical Computing, Vienna, Asutria. http://www.R-project.org/).

## Results

**Fig. 2:**
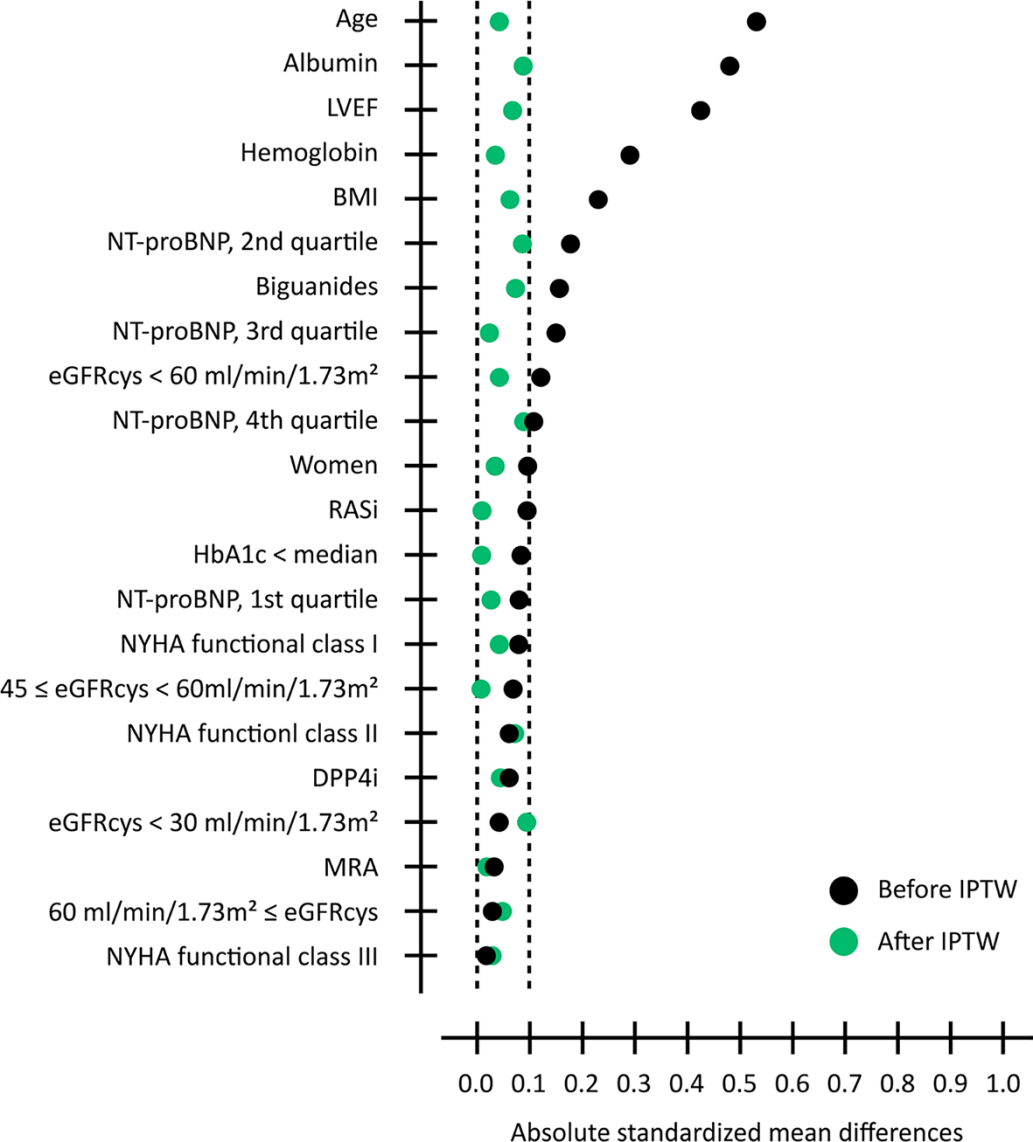
Distribution of standardized mean difference before and after inverse probability of treatment weighting. IPTW, inverse probability of treatment weighting; LVEF, left ventricular ejection fraction; BMI, body mass index; NT-proBNP, N-terminal pro-brain natriuretic peptide; eGFRcys, cystatine C-based estimated glomerular filtration rate; RASi, renin-angiotensin system inhibitors; HbA1c, glycated hemoglobin A1c; New York Heart Association; DPP4i, dipeptidyl peptidase 4 inhibitors; MRA, mineralocorticoid receptor antagonists.

One hundred forty-seven patients met the inclusion criterion, and 69 patients were excluded by the exclusion criteria. Thus, data for 81 patients were used for analyses as shown in Fig. 2.

### Baseline clinical characteristics

As shown in Table 1, patients receiving an SGLT2i tended to be younger than patients not receiving an SGLT2i and had a lower prevalence of ischemic heart disease than that in patients not receiving an SGLT2i, whereas age, sex and NYHA functional class were not significantly different between the two groups. LVEF was lower in patients receiving an SGLT2i than in patients not receiving an SGLT2i, resulting in a higher proportion of HFrEF patients in the SGLT2i-treated group. Plasma level of albumin was significantly higher and hemoglobin level tended to be higher in patients receiving an SGLT2i, but plasma NT-proBNP levels were comparable in the two groups. Biguanides was more frequently used in patients receiving an SGLT2i than in patients not receiving an SGLT2i, but the proportions of patients receiving an RASi, beta-blockers, MRA, DPP4i and loop diuretics were similar in the two groups.

**Table 1:** Baseline characteristics

	Missing	Non-SGLT2i	SGLT2i	p value
	n (%)	n = 52	n = 29	
Age, years		69 ± 12	65 ± 9	0.139
Women, n (%)		10 (19)	8 (28)	0.386
Height, cm		163 ± 9	163 ± 7	0.812
Weight, kg		64.3 ± 14.0	66.8 ± 17.3	0.489
Body mass index, kg/m^2^		24.0 ± 4.1	25.0 ± 5.3	0.353
Systolic blood pressure, mmHg		114 ± 16	112 ± 18	0.55
Diastolic blood pressure, mmHg		66 ± 10	69 ± 11	0.208
Heart rate, bpm		68 ± 12	68 ± 9	0.960
NYHA, n (%)				0.454
1		7 (14)	6 (21)	
2		24 (46)	15 (52)	
3		21 (40)	8 (28)	
Ischemic etiology, n (%)		25 (48)	9 (31)	0.14
Comorbidity, n (%)				
Hypertension		37 (71)	22 (76)	0.648
Dyslipidemia		38 (73)	20 (69)	0.694
LVEF, %		42.2 ± 2.1	35.1 ± 2.9	0.049
HFrEF, n (%)		26 (50)	21 (72)	0.050
Laboratory Data				
Albumin, mg/dl		3.5 ± 0.5	3.8 ± 0.5	0.014
Hemoglobin, mg/dl		12.5 ± 2.0	13.3 ± 2.2	0.118
eGFRcre, mL/min/1.73m^2^		55.3 ± 22.0	51.9 ± 20.6	0.503
eGFRcys, mL/min/1.73m^2^	8 (10)	56.8 ± 23.0	56.0 ± 24.5	0.890
NT-proBNP, pg/ml		2,909 ± 6,413	3,099 ± 8,053	0.907
Uric acid, mg/dl		6.0 ± 1.4	5.7 ± 1.8	0.551
Fasting blood glucose, mg/dl	2 (2)	112 ± 34	119 ± 39	0.433
Insulin, mIU/mL	3 (4)	8.1 ± 6.7	6.8 ± 5.4	0.371
HbA1c, %	1 (1)	6.8 ± 0.9	7.1 ± 1.0	0.234
Medication, n (%)				
RASi		30 (58)	20 (69)	0.317
Beta blocker		40 (77)	24 (83)	0.536
MRA		24 (46)	14 (48)	0.854
Loop diuretics		33 (63)	20 (69)	0.618
DPP4i		20 (38)	15 (52)	0.248
Biguanides		1 (2)	6 (21)	0.004
Insulin		7 (13)	6 (21)	0.396

Data are presented as mean ± standard deviation of the mean or number (with percentage). n, number of patients for whom the parameter was available. SGLT2, sodium-glucose cotransporter 2; NYHA, New York Heart Association; LVEF, left ventricular ejection fraction; HFrEF, heart failure with reduced ejection fraction; eGFRcre, creatinine-based estimated glomerular filtration rate; eGFRcys, cystatin C-based estimated glomerular filtration rate; NT-proBNP, N-terminal pro-brain natriuretic peptide; HbA1c, glycated hemoglobin A1c; RASi, renin-angiotensin system inhibitor; MRA, mineralocorticoid receptor antagonist; DPP4i, dipeptidyl peptidase 4 inhibitor

### Differences in plasma amino acid profiles between patients receiving SGLT2i and those not receiving SGLT2i

Of essential amino acids, leucine and histidine levels tended to be higher in patients receiving an SGLT2i than in patients not receiving an SGLT2i, whereas plasma levels of non-essential amino acids were similar in the two groups (Table 2). Of amino acid metabolites other than essential amino acids and non-essential amino acids, plasma BAIBA, a non-proteinogenic amino acid also known as 3-aminoisobutyric acid or 3-amino-2-methyproponic acid, was detected in 77% of all patients (i.e., it was below the detection limit in 23% of the study subject) and the proportion of patients in whom plasma BAIBA was detected was higher in patients receiving an SGLT2i than in patients not receiving an SGLT2i (93% vs. 67%, p = 0.01). Plasma BAIBA concentrations in the present study were consistent with those in earlier studies (approximately 794 to 4147 nM) [30, 31], indicating reasonable accuracy in our analyses. Analyses in patients in whom plasma BAIBA was detected showed that plasma BAIBA concentration was significantly higher in patients receiving an SGLT2i than in patients not receiving an SGLT2i (Table 2).

**Table 2:** Amino acid concentrations

	Normal range (nmol/ml)	Non-SGLT2i n = 52	SGLT2i n = 29	p
Essential amino acid				
Valine	158.4–287.7	248.1 ± 46.9	261.4 ± 58.6	0.268
Leucine	80.9–154.3	137.1 ± 28.6	150.8 ± 42.7	0.088
Isoleucine	41.3–84.9	81.6 ± 17.4	86.3± 28.6	0.356
Lysine	118.7–257.0	229.8 ± 53.6	231.6 ± 53.5	0.883
Methionine	19.2–32.7	26.8 ± 7.4	28.9 ± 9.4	0.265
Phenylalanine	45.7–76.5	69.6 ± 10.8	71.8 ± 12.6	0.400
Threonine	89.2–205.0	136.4 ± 35.5	139.9 ± 46.2	0.701
Tryptophan	41.4–65.5	45.8 ± 8.95	46.9 ± 9.3	0.601
Histidine	67.9–97.1	73.7 ± 12.4	79.0 ± 13.7	0.079
Tyrosine	50.2–82.6	69.5 ± 14.6	69.3 ± 15.1	0.961
Non-essential amino acid				
Glycine	153.2–362.1	237.0 ± 52.9	241.7 ± 55.6	0.708
Alanine	239.9 –510.2	367.5 ± 105.6	381.7 ± 91.8	0.546
Arginine	46.0–121.7	68.1 ± 21.9	65.1 ± 20.8	0.558
Cystine	36.5–56.0	67.0 ± 18.0	70.0 ± 20.3	0.494
Asparagine	40.8–76.5	62.2 ± 12.0	65.0 ± 12.1	0.332
Aspartic acid	< 3.2	4.13 ± 1.23, n = 50	4.16 ± 1.04, n = 28	0.922
Glutamine	488.2–733.1	566.8 ± 71.1	551.4 ± 103.5	0.431
Glutamic acid	10.8–44.4	68.7 ± 24.1	77.1 ± 26.6	0.151
Serine	91.5–161.8	134.4 ± 27.9	132.8 ± 36.3	0.815
Proline	89.6 –258.8	198.0 ± 65.5	202.1 ± 48.0	0.766
Other amino acid metabolites				
Taurine	35.2–70.0	65.9 ± 27.7	68.8 ± 18.3	0.620
Hydroxyproline	5.4 –18.2	12.3 ± 5.5	12.9 ± 5.0	0.605
Citrulline	20.4–44.8	41.8 ± 19.4	43.5 ± 18.8	0.694
β-aminoisobutyric acid (BAIBA)	< 3.7	4.56 ± 2.93, n = 35	6.76 ± 4.72, n = 27	0.028
α-Amino-n-butyric acid	11.0 –25.7	25.1 ± 8.7	27.7 ± 10.6	0.250
β-Alanine	< 7.7	7.81 ± 2.62	8.23 ± 2.59, n = 27	0.494
Monoethanolamine	6.0–10.7	8.50 ± 1.61, n = 48	9.26 ± 2.65, n = 26	0.131
Ornithine	43.2–95.7	111.1 ± 30.0	121.9 ± 45.7	0.207
1-Methylhistidine	< 12.8	13.6 ± 10.1	18.5 ± 15.8	0.088
3-Methylhistidine	2.9 –6.8	9.15 ± 7.24	11.82 ± 9.28	0.155

Data are presented as mean ± standard deviation of the mean or number (with percentage). n, number of patients for whom the parameter was available. SGLT2i, sodium-glucose cotransporter 2 inhibitor

### Independent association of elevated circulating β-aminoisobutyric acid level with SGLT2i use

To minimize the effects of selection bias in a retrospective study, two approaches were attempted. First, multivariate analyses in which BAIBA detection served as an outcome variable were performed. In multivariate logistic regression analyses that were adjusted for age and sex, SGLT2i use was independently associated with BAIBA detection (odds ratio: 7.59, 95% confidence interval: 1.56–36.90, Table 3). The independent association between BAIBA detection and SGLT2i use remained after inclusion of log BMI, HFrEF, ischemic etiology, log eGFRcys, log NT-proBNP, albumin, hemoglobin, and HbA1c in addition to age and sex into the logistic regression model (Table 3).

**Table 3:** Multivariate analyses by Cox-proportional hazards model

Model 1			Model 2			Model 3		
	OR (95% CI)	p		OR (95% CI)	p		OR (95% CI)	p
Age	1.04 (0.99–1.09)	0.12	Age	1.04 (0.99–1.09)	0.15	Age	1.04 (0.99–1.09)	0.12
Gender (Women)	1.41 (0.33–5.97)	0.64	Gender (Women)	1.41 (0.32–6.31)	0.65	Gender (Women)	1.43 (0.34–6.11)	0.63
SGLT2i, yes	7.59 (1.56–36.90)	0.01	SGLT2i, yes	7.59 (1.55–37.13)	0.01	SGLT2i, yes	7.13 (1.44–35.30)	0.02
			Log BMI	1.01 (0.03–38.34)	0.99	HFrEF	1.37 (0.45–4.16)	0.58

Model 4			Model 5			Model 6		
	OR (95% CI)	p		OR (95% CI)	p		OR (95% CI)	p
Age	1.04 (0.99–1.09)	0.12	Age	1.05 (0.99–1.11)	0.08	Age	1.10 (0.99–1.10)	0.13
Gender (Women)	1.36 (0.28–6.48)	0.70	Gender (Women)	1.52 (0.35–6.54)	0.58	Gender (Women)	1.48 (0.33–6.62)	0.61
SGLT2i, yes	7.52 (1.54–36.79)	0.01	SGLT2i, yes	8.10 (0.35–6.54)	0.01	SGLT2i, yes	7.70 (1.58–37.59)	0.01
Ischemic, yes	0.92 (0.27–3.11)	0.90	Log eGFRcys	2.26 (0.65–7.87)	0.20	Log NT-proBNP	0.94 (0.59–1.48)	0.79

Model 7			Model 8			Model 8		
	OR (95% CI)	p		OR (95% CI)	p		OR (95% CI)	p
Age	1.06 (1.00-1.16)	0.04	Age	1.05 (0.99–1.11)	0.06	Age	1.04 (0.99–1.08)	0.15
Gender (Women)	2.36 (0.48–11.44)	0.27	Gender (Women)	1.85 (0.41–8.46)	0.43	Gender (Women)	1.42 (0.34-6.00)	0.63
SGLT2i, yes	6.32 (1.25–31.8)	0.03	SGLT2i, yes	7.39 (1.49–36.65)	0.01	SGLT2i, yes	8.54 (1.71–42.68)	< 0.01
Albumin	4.82 (1.06–21.97)	0.04	Hemoglobin	1.23 (0.90–1.11)	0.20	HbA1c	0.75 (0.42–1.35)	0.34

SGLT2, sodium-glucose cotransporter 2; BMI, body mass index; HFrEF, heart failure with reduced ejection fraction; eGFRcys, cystatine C-based estimated glomerular filtration rate; NT-proBNP, N-terminal pro-brain natriuretic peptide; HbA1c, glycated hemoglobin A1c

**Fig. 3:**
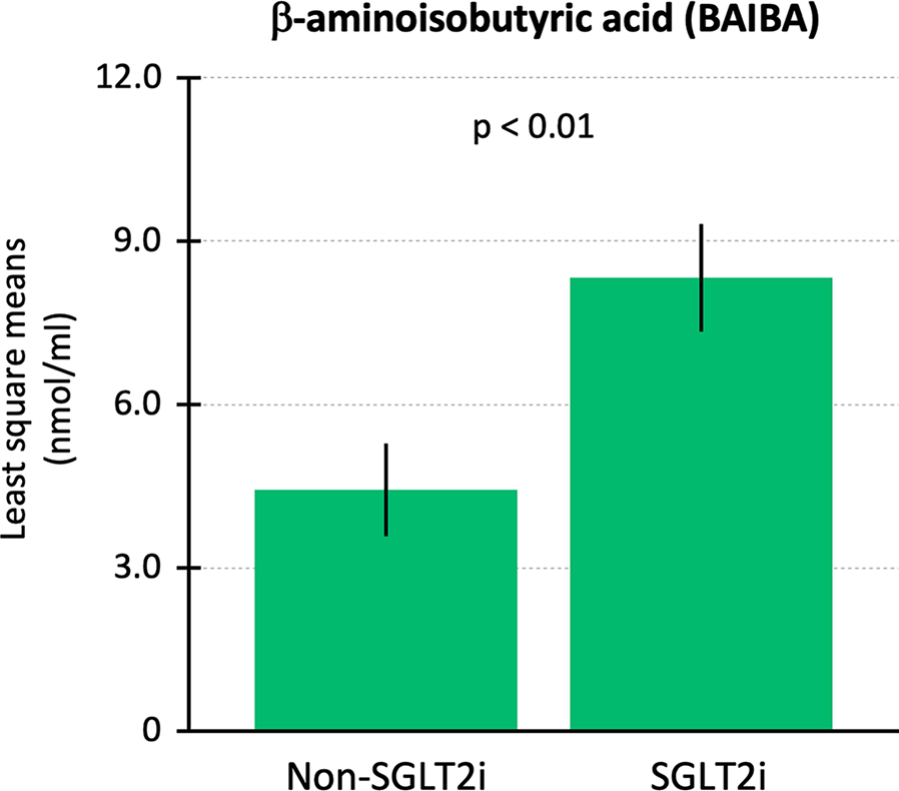
The least squares means of plasma β-aminoisobutyric acid (BAIBA) concentration after inverse probability of treatment weighting. SGLT2i, sodium-glucose cotransporter inhibitor; BAIBA, β-aminoisobutyric acid

Next, the differences in baseline characteristics between patients receiving an SGLT2i and patients not receiving an SGLT2i were controlled by using an IPTW-weighted analysis in 62 HF patients in whom plasma BAIBA was detected (Fig. 2). After IPTW, the SMDs of all covariates were less than 0.1, indicating that baseline differences in incorporated covariates were substantially improved as shown in Fig. 2. The least squares means of plasma BAIBA concentration were significantly higher in patients receiving an SGLT2i than in patients not receiving an SGLT2i (8.33 ± 0.99 vs. 4.43 ± 0.85 nmol/ml, p < 0.01, Fig. 3).

## Discussion

There have been several studies showing alterations in circulating metabolites including amino acids caused by treatment with an SGLT2i [32–35], but there have been no study focusing on BAIBA, a non-proteinogenic amino acid also known as 3-aminoisobutyric acid or 3-amino-2-methyproponic acid. In the present study, the proportion of patients in whom plasma BAIBA was detected was higher in patients receiving an SGLT2i than in patients not receiving an SGLT2i. Analyses in patients in whom plasma BAIBA was detected showed that plasma BAIBA concentration was significantly higher in patients receiving an SGLT2i than in patients not receiving an SGLT2i. After the effects of selection bias in a retrospective study had been minimized, SGLT2i use was significantly associated with increase in circulating BAIBA. To our knowledge, this study is the first study showing a close association between SGLT2 inhibition and upregulation of circulating BAIBA.

### Potential link between BAIBA and cardiovascular/renal protective effects of SGLT2 inhibition

**Fig. 4:**
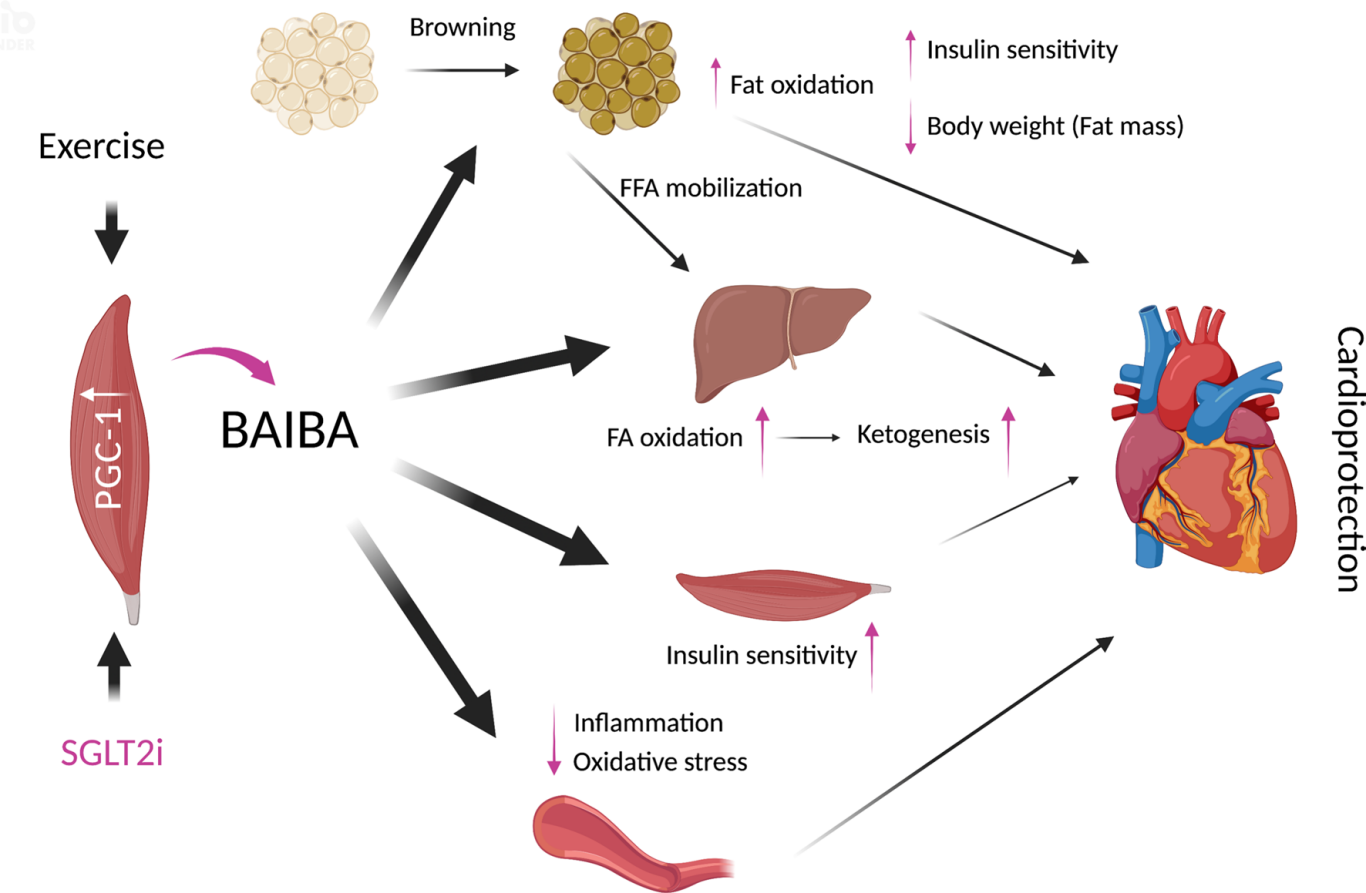
A working hypothesis: Circulating β-aminoisobutyric acid (BAIBA) mediates SGLT2i-induced cardioprotection. This figure was created with BioRender.com. SGLT2i, sodium-glucose cotransporter inhibitor; BAIBA, β-aminoisobutyric acid; FFA, free fatty acid; FA, fatty acid

Physical activity or exercise undoubtedly exerts benefits for life style-related disorders, which are at least partly mediated by myokines, cytokines and other peptides produced and released by muscle fibers [36, 37]. Several molecules such as IL-6, an extensively investigated myokine, have been shown to function as mediators of exercise-induced organ protection, one of which is BAIBA [36–38]. Plasma BAIBA concentrations have been shown to be increased by acute or chronic exercise in human though a conflicting data have also been reported [30, 38, 39]. Interestingly, the beneficial effects of an SGLT2i seem to be at least partly similar to reported functions of BAIBA (Fig. 4). First, a pioneering study by Roberts LD, et al. showed that BAIBA induced browning of white adipocytes and enhanced β-oxidation in hepatocytes both in vitro and in vivo through a PPARα-dependent pathway, which mimics the effects of forced expression of transcriptional coactivator peroxisome proliferator-activated receptor-gamma coactivator-1α (PGC-1α), a transcriptional factor that mediates favorable effects of exercise [38]. Furthermore, BAIBA administration reduced body weight gain and improved glucose tolerance in diet-induced obese mice [38]. In humans, plasma BAIBA concentration is inversely correlated with fasting glucose, insulin, the Homeostatic Model Assessment-Insulin Resistance, and triglycerides [38], i.e., BAIBA is closely associated with better metabolic profiles. These effects of BAIBA are mirrored by those of an SGLT2i: a large number of body composition analyses repeatedly revealed that treatment with an SGLT2i reduced fat mass in patients with DM [40], which was associated with improvement in metabolic profiles. Second, BAIBA treatment reduced production of proinflammatory cytokines and adhesion molecules induced by various stimuli and increased production of anti-oxidants [41–43], leading to suppression of atherosclerotic plaque formation. Anti-oxidative properties of an SGLT2i, a possible mechanism of cardiovascular/renal protection, through reduced production of free radicals and upregulation of anti-oxidants have been shown in multiple studies, though the mechanisms are not consistent and are possibly in an organ-specific fashion [44]. In addition, reduced expression of inflammatory markers such as IL-6, TNF-α and adhesion molecules by SGLT2 inhibition in a glucose concentration-independent fashion has been shown mainly in experimental studies [44, 45], though the association between BAIBA and activation of NLRP3 inflammasome, a known off-target of an SGLT2i, has not been investigated. Third, there is a hypothesis that elevated ketone concentration is attributable to SGLT2i-mediated protection from HF, though there are some debates [5, 14]. Ketones, which are known to be upregulated by SGLT2 inhibition in plasma, have been shown to provide ATP more efficiently than fatty acids and serve as an activator of protective molecular signaling [5, 14]. Increased production of free fatty acid by lipolysis and enhanced glucagon/insulin ratio are a possible primary mechanism of ketone production by SGLT2 inhibition [5, 14, 46], but the mechanisms by which SGLT2 inhibition upregulates ketone production remain poorly understood. BAIBA-induced enhancement of β-oxidation in hepatocytes, leading to ketone production, is an attractive candidate for the mechanism of SGLT2i-induced increase in circulating ketones. Finally, no increase in hypoglycemic episodes or their reduction by SGLT2i use has been proposed as a mechanism of reduced cardiovascular events by SGLT2i. Increased glucagon concentration by SGLT2 inhibition is a possible contributor, but this theory was questioned by results of a recent randomized, double-blind, crossover study using a hyperinsulinemic-hypoglycemic clamp [47]. Intriguingly, 3-hydroxyisobutyrate, an intermediate in catabolic reaction of valine leading to BAIBA production, is a known gluconeogenic substrate in isolated cortical tubules and hepatocytes [48], i.e., SGLT2i-induced valine catabolism, if any, may prevent hypoglycemia. Thus, several lines of evidence show that multiple effects of SGLT2 inhibition may be at least partly intervened by circulating BAIBA, leading to an attractive working hypothesis (Fig. 4): circulating BAIBA mediates SGLT2i-induced cardioprotection, though further studies including experimental studies are needed to determine them.

### Mechanisms of SGLT2i-induced BAIBA upregulation

SGLT2 inhibition-mediated glycosuria increases the net urinary caloric loss to approximately 200–300 kcal/day, promoting a fasting-like metabolic program including free fatty acid oxidation, glucogenesis, and ketogenesis, which is triggered by activation of AMP-activated protein kinase (AMPK) [3–5, 14, 49]. AMPK activates PGC-1α in SIRT1, a NAD+-dependent protein deacetylase, -dependent and -independent mechanisms, whereas it inhibits mTORC1 activity, compensating for the state of energy depletion [5, 49]. In addition, activation of the AMPK/PGC-1α pathway and inhibition of mTORC1 activity by SGLT2 inhibition has been shown to attenuate mitochondrial dysfunction, oxidative stress, and inflammation, where they promote autophagy [5, 49]. Intriguingly, BAIBA is released to circulation through a PGC-1α-dependent mechanism in myocytes, which is a mechanism of exercise-induced increase in circulating BAIBA [38]. There are several experimental studies showing enhancement in PGC-1α activity by treatment with an SGLT2i in the muscle and liver [49, 50], though there was a contradicted observation [51]. Collectively, the results indicate that an SGLT2i may serve as an exercise mimetic in addition to functioning as a starvation mimetic via PGC-1α-mediated increase in circulating BAIBA. However, further rigorous analyses are required to demonstrate the mechanism by which SGLT2i treatment enhances circulating BAIBA.

### Regulations and functions of two BAIBA enantiomers

Since BAIBA has a chiral center, there are two enantiomers of BAIBA in biological systems: D-BAIBA (R-BAIBA) and L-BAIBA (S-BAIBA), which are produced by distinct pathways [30, 52]. The predominant enantiomer in urine is D-BAIBA, but there are conflicting findings regarding the relative proportions of D- and L-BAIBA to total BAIBA in plasma [30, 52]. D-BAIBA is produced from thymine in the cytosol of the liver and kidney and is subsequently metabolized into methymalonate semialdehyde (MMS) in mitochondria, whereas valine is transaminated and oxidized to MMS in the mitochondria of muscle cells, leading to production of L-BAIBA, which is catalyzed by 4-aminobutyrate aminotransaminase (ABAT), i.e., L-BAIBA is a catabolic product of valine [30, 52]. The conversion of MMS to L-BAIBA by ABAT is known to be bidirectional. As a result, mitochondria are convergence points of the two BAIBA enantiomers and some L-BAIBA can be converted to D-BAIBA, and vice-versa, through MMS in the mitochondria [52]. However, the mechanism of systemic regulation of each BAIBA concentration remains to be elucidated. The conflicting findings regarding the relative proportions of D- and L-BAIBA may be explained by the presence of these shared pathways of BAIBA metabolism. Importantly, although both BAIBA enantiomers have been shown to be increased by exercise, the increase in L-BAIBA after exercise was significantly correlated with peak oxygen consumption, an index of aerobic exercise capacity [30], suggesting enhanced L-BAIBA release from muscle by exercise-induced upregulation of PGC-1α. In addition, a metabolomic analysis by Kappel BA, et al. showed that treatment with empagliflozin, an SGLT2i, restored HF-induced reduction in catabolism of branched chain amino acids (BCAA), an ATP source of failing hearts [53], suggesting that the myocardial availability of valine in blood was increased by SGLT2 inhibition, which may serve as a substrate for production of L-BAIBA. However, it remains unclear whether this is the case in HF patients with DM.

### Limitations

There are limitations in the present study. First, there might have been selection bias in the study subjects even after IPTW since this study was a retrospective, observational, and cross-sectional study using a small number of patients in a single center. Second, baseline BAIBA concentration has been shown to be modulated by a polymorphism of the alanine-glyoxylate aminotransferase 2 gene (rs37369) [30, 52], which was not analyzed in the present study. Third, the number of patients was insufficient for detection of differences among the groups with different HF etiologies and LVEF categories of HF (HFrEF and non-HFrEF). In addition, the involvement of other anti-diabetic agents in this hypothesis should be analyzed in the future. Especially, biguanides have been shown to be an activator of AMPK [54], theoretically leading to upregulation of circulating BAIBA. This hypothesis should be analyzed in the study including a large number of patients receiving biguanides. Fourth, comparative analyses of plasma amino acid profiles in HF patients and age-matched non-HF controls were not performed in the present study. Furthermore, circulating BAIBA concentration in non-diabetic HF patients receiving SGLT2i was not analyzed since an SGLT2i was not indicated for HF treatment in this study period. Importantly, concentrations of BCAA including valine, a substrate of BAIBA, were elevated in patients with T2DM [55], possibly resulting in differences between plasma BAIBA concentrations in T2DM patients and non-T2DM patients. Further detailed analyses according to clinical backgrounds are needed to confirm the results of the present study. Finally, race-dependent variation in plasma metabolic profiles has been shown in an earlier study [17]. The findings of the present study may not be extrapolated to other races with HF.

## Conclusion

SGLT2i use is closely associated with increased circulating BAIBA concentration in HF patients with T2DM. This study was a preliminary retrospective study, but the results provide novel insights into the mechanism of SGLT2i-induced cardioprotection.

## Data Availability

The datasets generated and/or analyzed during the current study are not publicly available because a research agreement from all authors is required for data sharing, but are available from the corresponding author on reasonable request.

## Abbreviations

SGLT2i: Sodium-glucose cotransporter 2 inhibitors
HF: Heart failure
T2DM: Type 2 diabetes mellitus
IPTW: Inverse probability of treatment weighting
BAIBA: b-aminoisobutyric acid
NT-proBNP: N-terminal pro-brain natriuretic peptide
HbA1c: Glycated hemoglobin A1c
HFrEF: Heart failure reduced ejection fraction
HFpEF: Heart failure preserved ejection fraction
EGFR: Estimated glomerular filtration rate
BMI: Body mass index
LVEF: Left ventricular ejection fraction
UPLC: Ultraperformance liquid chromatography
IQR: Interquartile range
PS: Propensity score
RASIs: Renin-angiotensin system inhibitors
MRA: Mineralocorticoid receptor antagonists
DPP4i: Dipeptidyl peptidase 4 inhibitors
SMD: Standardized mean difference
PGC-1α: Transcriptional coactivator peroxisome proliferator-activated receptor-gamma coactivator-1α
AMPK: AMP-activated protein kinase
MMS: Methymalonate semialdehyde
ABAT: 4-aminobutyrate aminotransaminase
BCAA: Branched chain amino acids
